# Bioactive Phenolics from Vinegar–Egg Accelerates Acute Wound Healing by Activation of Focal Adhesion and Mitogen-Activated Protein Kinase Signaling

**DOI:** 10.3390/nu17162584

**Published:** 2025-08-08

**Authors:** Taehoon Oh, Chan Hee Cho, Su Cheol Baek, Mun Seok Jo, Woo Bong Kang, Yun Seok Kang, Sung-Kyun Ko, Ki Hyun Kim

**Affiliations:** 1 Chemical Biology Research Center, Korea Research Institute of Bioscience and Biotechnology (KRIBB), Cheongju 28116, Republic of Korea; dhxogns10@kribb.re.kr; 2 College of Pharmacy, Chungbuk National University, Cheongju 28160, Republic of Korea; 3 School of Pharmacy, Sungkyunkwan University, Suwon 16419, Republic of Korea; whckswhcks@g.skku.edu (C.H.C.); schii513@daum.net (S.C.B.); anstjr920827@gmail.com (M.S.J.); 4 Crystal Insul Bio, Paju 10930, Republic of Korea; sjcbkemp@gmail.com (W.B.K.); ko2niyiru@naver.com (Y.S.K.); 5 KRIBB School of Bioscience, Korea University of Science and Technology (UST), Daejeon 34141, Republic of Korea

**Keywords:** vinegar–egg, 4-hydroxy-benzoic acid, vanillic acid, wound healing, focal adhesion kinase (FAK), mitogen-activated protein kinase (MAPK)

## Abstract

Background/Objectives: Vinegar–egg is a traditional health-promoting beverage prepared by soaking eggs in vinegar. While both eggs and vinegar are common dietary components with well-documented nutritional and pharmacological activities, eggs treated with vinegar have been rarely studied. This study aims to identify and characterize bioactive compounds in vinegar–egg and investigate their potential wound-healing activities. Methods: The vinegar–egg extract was analyzed using liquid chromatography–mass spectrometry (LC–MS) and column chromatography, including HPLC purification, which led to the isolation of four phenolic compounds. Results: These compounds were identified as 4-hydroxybenzoic acid (**1**), vanillic acid (**2**), methyl syringate (**3**), and leptosperin (**4**) using ESI-MS, UV, and NMR spectroscopic data. Among the isolates, 4-hydroxybenzoic acid (**1**) and vanillic acid (**2**) demonstrated wound-healing properties in mouse embryonic fibroblast (MEF) cells. None of the compounds, 4-hydroxybenzoic acid (**1**), vanillic acid (**2**), methyl syringate (**3**), or leptosperin (**4**), exhibited cytotoxicity in PC12, AGS, MEF, or MDA-MB-231 cells. Notably, 4-hydroxybenzoic acid (**1**) enhanced cell motility by 2.59-fold and cell invasion by 1.20-fold, while vanillic acid (**2**) increased cell motility by 2.69-fold and cell invasion by 1.23-fold. Western blot analysis revealed that treatment with 4-hydroxybenzoic acid (**1**) and vanillic acid (**2**) increased the phosphorylation of focal adhesion kinase (p-FAK) and matrix metalloproteinase 2 (MMP-2). Furthermore, both compounds elevated the phosphorylation of p38, a key regulator in wound-healing pathways. Conclusions: These findings demonstrate that 4-hydroxybenzoic acid (**1**) and vanillic acid (**2**) accelerate wound healing through the activation of focal adhesion and mitogen-activated protein kinase (MAPK) signaling pathways. These results highlight vinegar–egg as a promising therapeutic candidate for wound healing.

## 1. Introduction

Wound healing involves coordinated interactions among diverse cell types, signaling molecules, and the extracellular matrix [1,2]. It proceeds by primary intention, which involves closure of wound edges by suturing, fibroblast driven proliferation, collagen deposition, angiogenesis, and epithelialization [3,4,5,6,7,8,9,10], and by secondary intention, in which open wounds are repaired through chemotactic recruitment of immune cells for debris clearance, growth factor secretion, and epidermal migration [3,11,12,13,14,15]. Together, these mechanisms restore tissue integrity [16,17].

The mitogen-activated protein kinase (MAPK) pathway is central to wound healing, being activated by growth factors and cytokines such as epidermal growth factor (EGF) and tumor necrosis factor-alpha (TNF-α) [12,18,19,20,21,22]. During the inflammatory stage, MAPK signaling regulates cell proliferation, differentiation, and survival by phosphorylating downstream targets in fibroblasts, endothelial cells, and other repair-related cell types [9,13,23,24,25,26]. Matrix metalloproteinase 2 (MMP2; gelatinase A), which is also induced early in healing, degrades collagen, elastin, and fibronectin to enable matrix remodeling [27,28,29,30]. Moreover, MAPK and MMP2 pathways are tightly interconnected: MAPK controls MMP2 expression and activity, while MMP2 supports MAPK-mediated cell migration and proliferation [8,31,32,33]. Together, these coordinated mechanisms underscore the therapeutic potential of targeting MAPK signaling and MMP2 modulation in chronic-wound treatment.

Eggs are one of the few foods consumed universally across the globe. They are rich in nutrients, with high protein content and amino acid compositions similar to those of human proteins. Eggs are considered a source of high-quality (complete) protein and contain numerous biologically active ingredients. Numerous studies have demonstrated that eggs possess a variety of health-promoting and medicinal properties, including antifungal, antihypertensive, antitumor, antioxidant, brain-enhancing, and immune-regulating effects [34,35,36,37,38,39]. Vinegar, a food product derived from microbial fermentation, is a common, widely used, and economical item in daily life. It is not only utilized to enhance the flavor of food and prevent spoilage but is also consumed in some countries as a health-promoting beverage [40]. With increasing research on vinegar, it has been shown to provide numerous health benefits. Its applications have expanded from a traditional condiment to a component in various food types, such as meals, beverages, and healthcare products [40]. While eggs are often consumed after thermal processing, a traditional health-promoting beverage known as vinegar–egg is produced in some Asian countries, including China and Korea, by soaking eggs in vinegar. The history of vinegar–egg dates back to the Han Dynasty, where it was used as a remedy for throat inflammation and voice loss. It continues to be consumed as a health-promoting beverage in these regions [38]. This unique beverage exhibits several pharmacological activities including antioxidant, immunomodulatory, and anti-inflammatory effects, as well as angiotensin-converting enzyme (ACE) inhibitory activity [38,41,42,43], indicating that treating eggs with vinegar holds significant potential as a non-thermal processing method that maximizes the nutritional preservation of eggs.

Previous studies have suggested some potential bioactivities of vinegar–egg; however, its wound-healing effects have not yet been explored. As part of our ongoing efforts in natural product discovery to identify bioactive ingredients from diverse natural resources [44,45,46,47,48], we conducted a chemical analysis to isolate and characterize potent bioactive compounds from vinegar–egg and evaluate its potential wound-healing properties. Although vinegar–egg comprises a complex mixture of proteins, peptides, amino acids, and other organic acids, the present study aimed to explore the bioactive potential of small molecules, particularly phenolic compounds—given their well-documented wound-healing and related biological activities [49]. The chemical investigation of vinegar–egg, utilizing liquid chromatography–mass spectrometry (LC–MS) and column chromatography, including HPLC purification, resulted in the isolation of four phenolic compounds (**1**–**4**). The chemical structures of these compounds were elucidated through nuclear magnetic resonance (NMR) spectroscopy, as well as UV and MS data obtained from LC–MS analysis. The wound-healing properties of the isolated compounds were assessed in mouse embryonic fibroblast (MEF) cells. In this study, we detail the process of isolating and structurally characterizing compounds **1**–**4** from vinegar–egg and investigate the molecular mechanisms underlying their wound-healing activities.

## 2. Materials and Methods

### 2.1. General Experimental Procedures

The detailed information is available in Supplementary Materials.

### 2.2. Preparation of Vinegar–Egg

Fresh chicken eggs (average mass: 45–55 g) and rice vinegar (9°) were purchased from a local market in Korea and used within 24 h of purchase. The eggs were washed with water, sterilized by wiping the shell with 95% ethanol, and then air-dried. The prepared egg samples were mixed with rice vinegar at a 1:1 weight ratio (eggs/vinegar) and kept at room temperature for 7 days. After the eggshell was completely dissolved, the solution was filtered through sterile gauze. The resulting filtrate was further incubated at room temperature for an additional 3 days. The final vinegar–egg solution was stored at −20 °C in a freezer until use.

### 2.3. Extraction and Isolation

The vinegar–egg solution was first suspended in 700 mL of distilled water and then sequentially partitioned with *n*-hexane, dichloromethane (CH_2_Cl_2_), ethyl acetate (EtOAc), and *n*-butanol (*n*-BuOH), using three 700 mL extractions for each solvent. This process yielded 0.6 g, 0.6 g, 0.7 g, and 2.8 g of the respective fractions. LC/MS analysis indicated that the major phenolic compounds were primarily distributed in the CH_2_Cl_2_, EtOAc, and *n*-BuOH-soluble fractions. The CH_2_Cl_2_-soluble fraction (0.6 g) was subjected to silica gel chromatography (230–400 mesh) using a CH_2_Cl_2_-MeOH-H_2_O solvent gradient (50:1–1:1:0.2, *v*/*v*), yielding four subfractions (OTC1–OTC4). From OTC1 (25 mg), compound **3** (0.7 mg, *t*_R_ = 26.0 min, 0.117%) was isolated via semi-preparative reversed-phase HPLC (Phenomenex Luna Phenyl-hexyl 100 Å column, 250 × 10 mm i.d., 10 μm) under an isocratic solvent system of 29% CH_3_CN/H_2_O at a flow rate of 2 mL/min. The EtOAc-soluble fraction (0.7 g) was subjected to silica gel chromatography (230–400 mesh) using a CH_2_Cl_2_-MeOH solvent gradient (50:1–1:1, *v*/*v*), yielding five subfractions (OTE1–OTE5). From OTE2 (56 mg), compound **1** (1.8 mg, *t*_R_ = 20.0 min, 0.257%) and compound **2** (0.8 mg, *t*_R_ = 23.0 min, 0.114%) were purified using semi-preparative reversed-phase HPLC on Phenomenex Luna Phenyl-hexyl 100 Å column (250 × 10 mm i.d., 10 μm particle size) under an isocratic mobile phase of 17% CH_3_CN/H_2_O at a flow rate of 2 mL/min. The *n*-BuOH-soluble fraction (2.8 g) was fractionated over silica gel (230–400 mesh) using a methanol–water gradient system (2:8–8:2, *v*/*v*), yielding five subfractions (OTB1–OTB5). From OTB4 (24 mg), compound **4** (0.6 mg, *t*_R_ = 19.0 min, 0.021%) was isolated via semi-preparative reversed-phase HPLC (Phenomenex Luna Phenyl-hexyl 100 Å column, 250 × 10 mm i.d., 10 μm) under an isocratic solvent system of 15% CH_3_CN/H_2_O at a flow rate of 2 mL/min. The isolated phenolic compounds (**1**–**4**) were used without any chemical modification in all subsequent in vitro biological assays.

### 2.4. Reagents and Antibodies

The HaCaT cell line (immortalized human keratinocytes, #300493) and MDA-MB-231 (human breast cancer, #300275) were acquired from Cytion (Eppelheim, Germany). The AGS (human gastric adenocarcinoma, #CRL-1739), HCT116 (human colorectal carcinoma, #CCL-247), A549 (human lung carcinoma, #CCL-185), MRC-5 (human lung fibroblasts, #CCL-171), MEF (mouse embryonic fibroblasts, #SCRC-1040), and PC12 (rat adrenal medulla pheochromocytoma, #CRL-1721) cell lines were acquired from the ATCC (American Type Culture Collection, Manassas, VA, USA). FBS (fetal bovine serum, S001-01) was sourced from Welgene (Gyeongsangbuk-do, Republic of Korea). Penicillin-streptomycin (P4333), paraformaldehyde (P6148), DMEM (Dulbecco’s modified Eagle’s medium, 10-013-CVRC), crystal violet (C6158), mitomycin C (M7949), phosphatase inhibitor cocktail 3 (P0044), epidermal growth factor (EGF; SRP6253), dimethyl sulfoxide (DMSO; 472301), and protease inhibitor cocktail (5056489001) were supplied by Sigma-Aldrich (St. Louis, MO, USA). M-PER buffer (#78501), chemiluminescent detection reagents used in this study, including SuperSignal West Pico (#34080) and SuperSignal West Femto Maximum Sensitivity substrate (#34095), were obtained from Thermo Fisher Scientific (Waltham, MA, USA). Matrigel (354234) and Transwell^®^ membranes (3422) were obtained from Corning (New York, NY, USA). The CytoX colorimetric assay (CYT3000) was obtained from LPS Solution (Deajeon, Republic of Korea). Primary antibodies against p-p38 MAPK(#4511), MMP2 (matrix metallopeptidase 2, #13132), and p38 MAPK (#9212) were supplied by Cell Signaling Technology (CST, Danvers, MA, USA), whereas antibodies against β-actin (#sc-47778), phosphorylated focal adhesion kinase (p-FAK, #sc-81493), and focal adhesion kinase (FAK, #sc-1688) were supplied by Santa Cruz Biotechnology (SCB, Dallas, TX, USA). The HRP-linked secondary antibodies, consisting of anti-mouse IgG (#7076) and anti-rabbit IgG (#7074), were acquired from CST.

### 2.5. Cell Culture

MEF, HaCaT, PC-12, AGS, MDA-MB-231, HCT116, A549, and MRC5 cells were grown at 37 °C with 5% CO_2_ in a humidified chamber. The culture medium consisted of DMEM supplemented with 10% FBS, along with penicillin (100 U/mL) and streptomycin (100 μg/mL).

### 2.6. Cell Viability and Cell Proliferation Assay

To evaluate cytotoxic effects, MEF, HaCaT, PC12, AGS, MDA-MB-231, HCT116, A549, and MRC5 cells were seeded at a density of 1.0 × 10^4^ cells per well in 96-well plates and incubated for 12 h. The cells were subsequently treated with compounds **1**–**4** at concentrations of up to 100 μM for 24 h. For proliferation analysis, MEF and HaCaT cells were plated at a density of 3.0 × 10^3^ cells per well and treated with compounds **1**–**4** at defined concentrations of 25, 50, and 100 μM for 24, 48, and 72 h. At each time point, the culture medium was replaced with CytoX reagent, followed by a 1 h incubation. Absorbance was recorded at 450 nm using a microplate reader (SpectraMax 190, Molecular Devices, San Jose, CA, USA), and cell viability and proliferation rates were calculated relative to untreated controls.

### 2.7. Scratch Wound Healing Assay

For the scratch wound healing assay, MEF and HaCaT cells were seeded into 24-well plates at a density of 6.0 × 10^4^ cells per well and incubated for 12 h to allow adherence. A uniform linear wound was generated at the center of each well using a sterile 200 μL pipette tip. To suppress cell proliferation, 10 μg/mL mitomycin C was applied for 3 h prior to compound treatment. Cells were then exposed to compounds **1**–**4** at final concentrations of 50 and 100 μM for 24 h. Following incubation, cells were fixed in 4% paraformaldehyde for 15 min and stained with 0.2% crystal violet. Images were acquired using a ZEISS microscope (Jena, Germany), and wound closure was quantitatively analyzed using ImageJ software (v1.53k, NIH, Bethesda, MD, USA).

### 2.8. Transwell Chamber Invasion Assay

Cell invasion was assessed using a Transwell^®^ system equipped with 6.5 mm inserts containing 8.0 μm pores (Corning, #3422, USA). MEF cells were seeded at a density of 6.0 × 10^4^ cells per well onto the Matrigel-coated upper chamber in 200 μL of serum-free medium supplemented with compounds **1**–**4**. The lower chamber was filled with DMEM containing FBS to serve as a chemoattractant. After a 24 h incubation period, non-invading cells on the upper membrane surface were carefully removed. The cells that had migrated through the membrane were fixed in 4% paraformaldehyde for 15 min, stained with 0.2% crystal violet, and visualized using a ZEISS microscope (Germany).

### 2.9. Time-Lapse Cell Tracking Analysis

For live cell migration tracking, MEF cells were seeded at a density of 9.0 × 10^3^ cells per 100 μL in DMEM lacking fetal bovine serum and supplemented with 2% Matrigel, using μ-Slide I chambers (Ibidi, #80106). After a 3 h pre-incubation period, 900 μL of DMEM containing 10% FBS was gently added to each well, followed by overnight incubation. Cells were subsequently treated with 100 μM of compounds **1**–**4** and immediately transferred to a HoloMonitor M4 imaging system (phase-holographic cytometry) maintained at 37 °C. Time-lapse images were acquired at 15 min intervals over a 12 h period, and cell motility was quantified using HoloStudio M4 analysis software (v2.6.4; Hstudio M4, Sun Valley, CA, USA).

### 2.10. Western Blot Analysis

Total protein extracts from MEF cells were prepared by lysing the cells on ice using cold M-PER buffer supplemented with both protease and phosphatase inhibitors. Lysates were clarified by centrifugation at 13,500 rpm for 15 min at 4 °C, and the supernatants were subjected to separation on 10% SDS-polyacrylamide gels. Proteins were subsequently transferred to nitrocellulose membranes with a pore size of 0.2 μm (Bio-Rad, #162-0112). Membranes were incubated with a blocking solution of 5% non-fat dry milk in tris-buffered saline containing 0.1% Tween-20 for 1 h at ambient temperature. Following blocking, membranes were incubated with the relevant primary antibodies overnight at 4 °C, then washed and treated with HRP-linked secondary antibodies (anti-mouse or anti-rabbit IgG, diluted 1:5000) for 2 h. Visualization of immunoreactive bands was performed using chemiluminescent substrates (SuperSignal West Pico or West Femto, Thermo Fisher Scientific).

### 2.11. Statistical Analysis

All results are expressed as the mean ± standard error of the mean (SEM), based on a minimum of three biologically independent replicates. Statistical analysis was conducted using GraphPad Prism software (v9.5.1; GraphPad Software, San Diego, CA, USA). Comparisons with the DMSO-treated control group were considered statistically significant at * *p* < 0.05, ** *p* < 0.01.

## 3. Results and Discussion

### 3.1. Isolation and Structure Elucidation of Compounds

**Figure 1:**
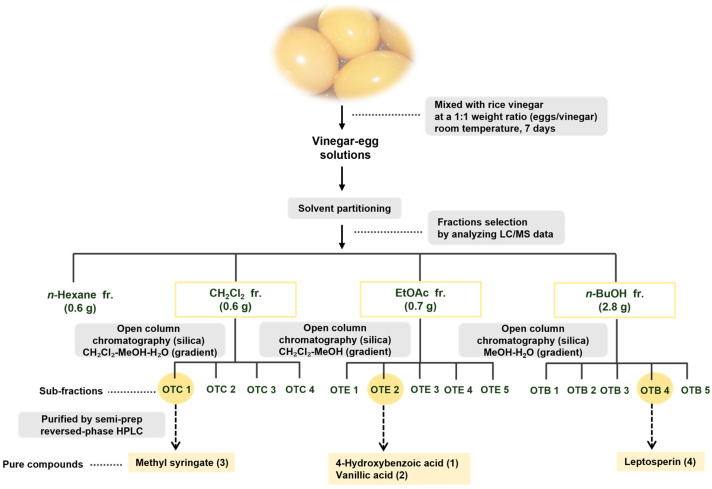
The separation scheme of compounds **1**–**4**.

**Figure 2:**
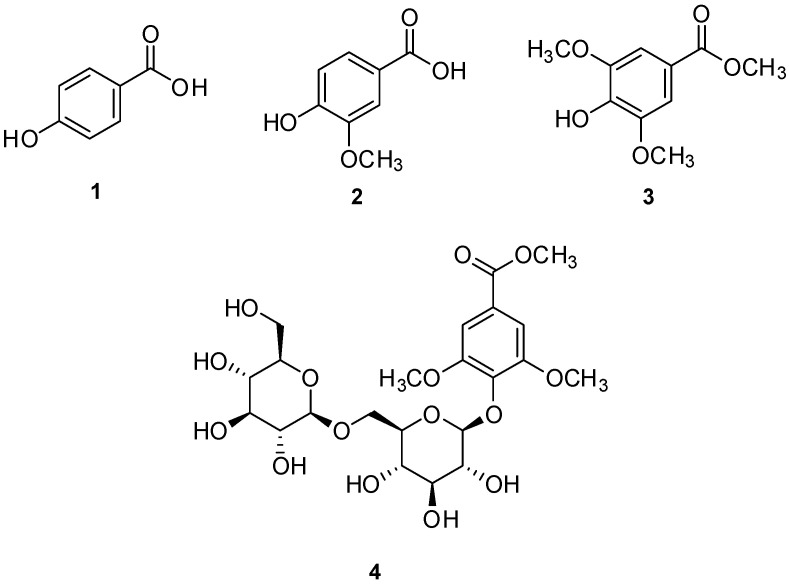
Structures of compounds **1**–**4** isolated from vinegar–egg. 4-Hydroxybenzoic acid (**1**), vanillic acid (**2**), methyl syringate (**3**), and leptosperin (**4**).

The prepared vinegar–egg solutions were suspended in distilled water and sequentially partitioned with four organic solvents of increasing polarity: *n*-hexane, CH_2_Cl_2_, EtOAc, and *n*-BuOH, yielding four respective fractions (Figure 1). LC–MS analysis, supported by a custom-built UV spectral library, revealed that the major phenolic constituents were concentrated in the CH_2_Cl_2_-, EtOAc-, and *n*-BuOH-soluble fractions. These fractions were further purified using a combination of open column chromatography and semi-preparative HPLC, resulting in the isolation of four phenolic compounds, designated as compounds **1**–**4** (Figure 1). Structural identification of these compounds was achieved by comparing their NMR spectroscopic data with literature values and LC–MS analysis (Figures S1–S8), confirming them as 4-hydroxybenzoic acid (**1**) [50], vanillic acid (**2**) [51], methyl syringate (**3**) [52], and leptosperin (**4**) [53] (Figure 2).

### 3.2. Effects of Compounds ***1**–**4*** on Cytotoxicity

**Figure 3:**
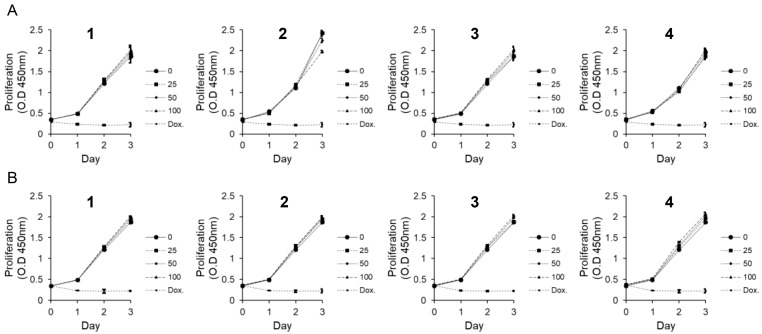
Effects of compounds **1**–**4** on the proliferation. Cell proliferation was evaluated in (**A**) MEF and (**B**) HaCaT cells following treatment with compounds **1**–**4** at concentrations of 0, 25, 50, and 100 μM for 24, 48, and 72 h. Cells were seeded in 96-well plates (3 × 10^3^ cells/well). Doxorubicin (5 μM) served as a positive control. Data are presented as mean ± SEM (*n* = 3). Abbreviations: OD, optical density; Dox, doxorubicin.

To investigate the potential cytotoxicity of compounds **1**–**4** in the context of wound healing, eight cell lines (PC-12, AGS, MDA-MB-231, HCT116, A549, MRC5, HaCaT, and MEF) were treated with the compounds at concentrations ranging from low doses up to 100 μM for 24 h. Cell viability was assessed using the CytoX colorimetric assay following treatment with compounds **1**–**4** at concentrations up to 100 μM, and no apparent cytotoxic effects were observed in AGS, MDA-MB-231, HCT116, A549, MRC5, HaCaT, MEF, and PC12 cells under the tested conditions. To further examine whether compounds **1**–**4** influence cellular proliferation, MEF and HaCaT cells were treated with 25, 50, and 100 μM of each compound and monitored over 24, 48, and 72 h. No significant changes in proliferation were detected compared to the DMSO control, indicating that the compounds did not adversely affect cell growth at the tested concentrations (Figure 3).

### 3.3. Effect of Compounds ***1**–**4*** on MEF Cells Motility

**Figure 4:**
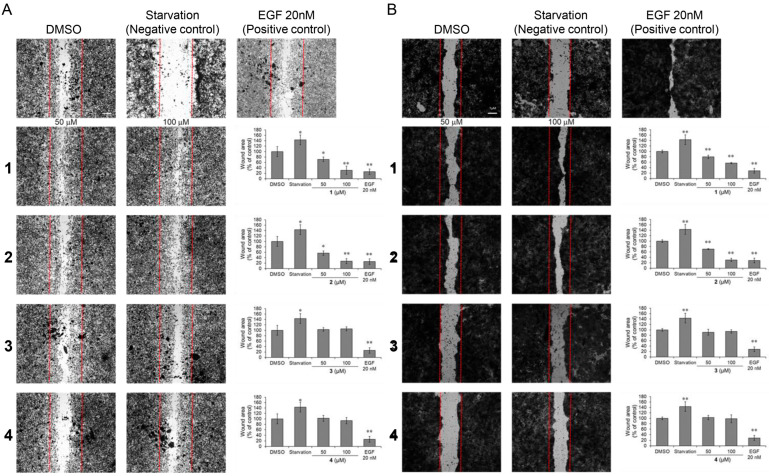
Effects of compounds **1**–**4** on cell migration in MEF cells. (**A**) MEF and (**B**) HaCaT cells were seeded in 24-well plate (6.0 × 10^4^ cells/well) and incubated with compounds **1**–**4** at 50 and 100 μM for 24 h. Epidermal growth factor (EGF, 20 nM) was used as a positive control. Cell migration was quantified using a scratch wound healing assay by measuring the area and width of the wound gap. Images were captured using a 4× objective lens, and scale bars indicate 1 mm. Data are expressed as mean ± SEM from three independent experiments (*n* = 3; * *p* < 0.05, ** *p* < 0.01 vs. DMSO-treated group).

**Figure 5:**
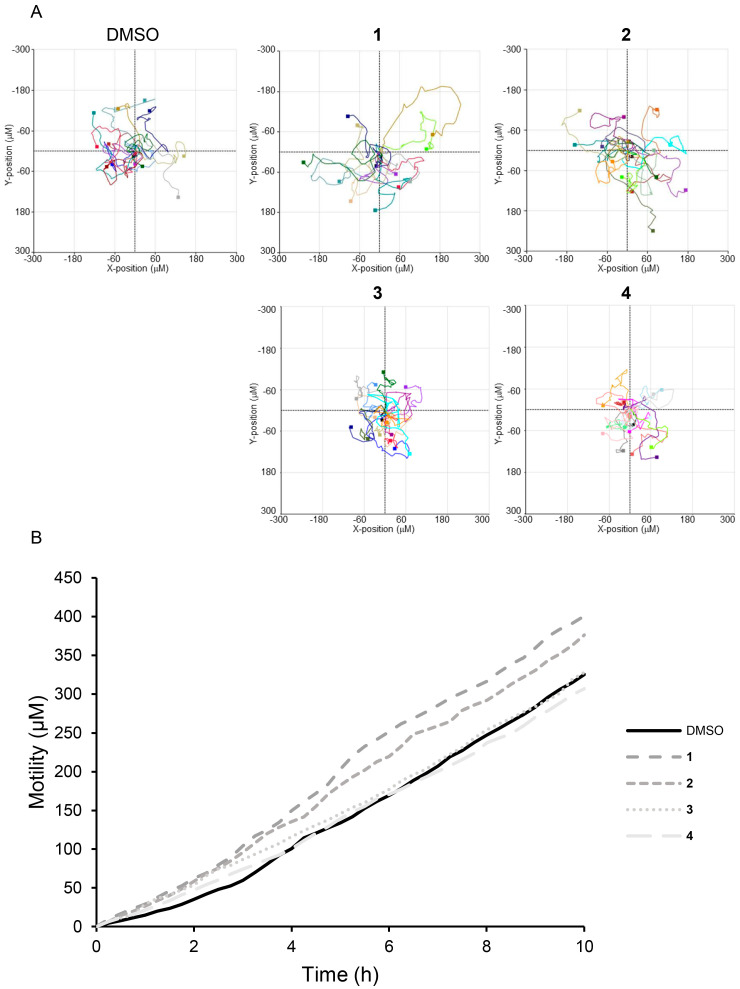
Effects of compounds **1**–**4** on the acceleration of cell migration in MEF cells. (**A**) The holographic recordings of live MEF cells treated with 100 μM compounds **1**–**4** were monitored every 15 min for 12 h. (**B**) The average of motility (μm) was analyzed using the HoloStudio software.

We performed a scratch wound healing assay to evaluate the wound closure effects of compounds **1**–**4**. Treatment with 100 μM of compounds **1**–**4** and EGF enhanced wound closure in MEF cells by 2.59-, 2.69-, 1.05-, 0.93-, and 2.72-fold, respectively, compared to DMSO-treated cells (Figure 4A). Treatment with 100 μM of compounds **1**–**4** and EGF enhanced wound closure in HaCaT cells by 1.76-, 3.27-, 1.07-, 1.02-, and 3.51-fold, respectively, compared to DMSO-treated cells (Figure 4B). Live cell analysis using the HoloMonitor M4, which tracks individual cells to monitor movement and migration patterns, confirmed that cells treated with compounds **1**–**4** exhibited enhanced motility. Cell migration was assessed to determine the distance and direction of cell movement. Matrigel was placed inside the chamber of a μ-Slide I to prevent medium circulation, while medium containing FBS was placed outside the chamber. Compared to DMSO, compounds **1** and **2** exhibited an increased tendency to move in different directions (Figure 5A). Compounds **1**–**4** moved 1.23-, 1.15-, 1.00-, and 0.94-fold faster than the DMSO-treated control group, respectively (Figure 5B). The complete cell motility video is provided in Supplementary Data (Videos S1–S5). These results suggest that the compounds **1**–**4** may influence cell migration and wound healing by promoting cell motility without increasing cell numbers.

### 3.4. Effect of 4-Hydroxy-Benzoic Acid (***1***) and Vanillic Acid (***2***) on MEF Cells Invasion

**Figure 6:**
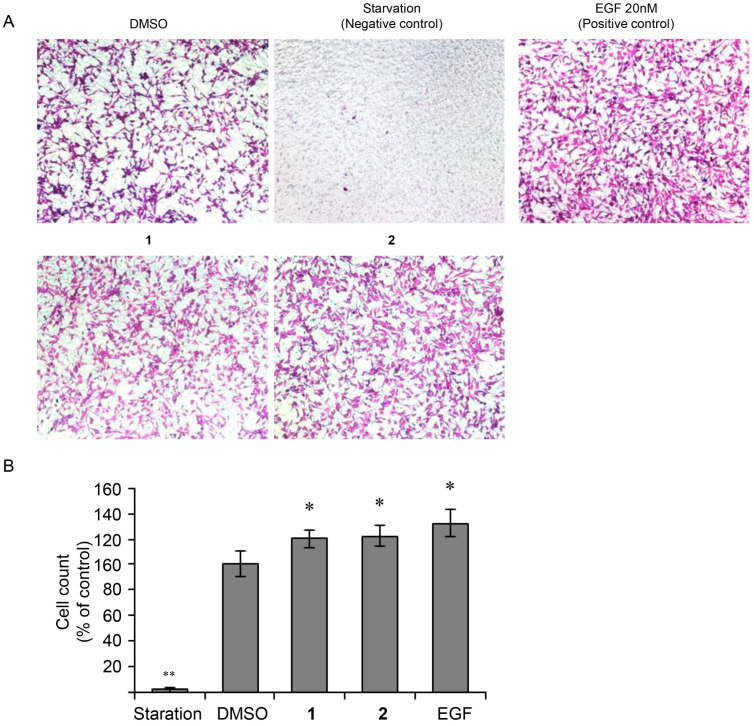
Matrigel invasion assay of MEF cells using a Transwell system. (**A**) Cells were seeded on a Transwell insert plate (2.5 × 10^4^ cells/well) and treated with 100 μM compounds **1** and **2** for 24 h. (**B**) Graph showing the invasion assay, including the measurement of cell numbers. Data are expressed as mean ± SEM from three independent experiments (*n* = 3; * *p* < 0.05, ** *p* < 0.01 vs. DMSO-treated group).

To confirm the effects of 4-hydroxybenzoic acid (**1**) and vanillic acid (**2**) on cell invasion, we performed a Transwell chamber invasion assay. Based on the assay results, treatment with 100 μM of 4-hydroxybenzoic acid (**1**) and vanillic acid (**2**), and EGF enhanced MEF cell invasion by 1.20-, 1.23-, and 1.32-fold, respectively, compared to DMSO-treated cells (Figure 6). These results indicate that while cell mobility was enhanced, there was no increase in cell number, suggesting that the tested compounds **1** and **2** may influence cell migration and wound healing processes. Specifically, 4-hydroxybenzoic acid (**1**) and vanillic acid (**2**) may enhance cell mobility by promoting invasion ability, thereby facilitating wound closure.

### 3.5. Acceleration of FAK and MAPK Phosphorylation in MEF Cells Following Treatment with 4-Hydroxy-Benzoic Acid (***1***) and Vanillic Acid (***2***)

**Figure 7:**
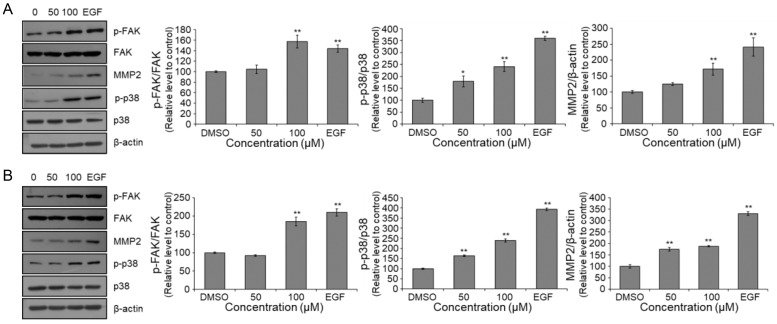
Involvement of the FAK pathway in MMP2 and MAPK expression and promotion of cell migration. MEF Cells were seeded in 6-well plates (1.5 × 10^5^ cells/well) and treated for 24 h with DMSO (0.1%, *v*/*v*), (**A**) 4-hydroxy-benzoic acid (**1**) or (**B**) vanillic acid (**2**) at 50 and 100 μM for 24 h. The protein levels of phosphorylated and total FAK and p38 MAPK (p-FAK/FAK and p-p38/p38 MAPK) were analyzed by Western blotting. To confirm equivalent protein amounts, β-actin was employed as a reference protein. All values are expressed as mean ± SEM (*n* = 3; * *p* < 0.05, ** *p* < 0.01 compared with the DMSO control group).

To further elucidate the potential mechanisms of action of 4-hydroxy-benzoic acid (**1**) and va-nillic acid (**2**), we examined their effects on MAPK phosphorylation. Our findings indicate that treatment with these compounds increased the phosphorylation of FAK and p38 in MEF cells (Figure 7). Additionally, MMP2 expression was upregulated in MEF cells. Collectively, these results suggest that the activation of MAPK signaling by 4-hydroxy-benzoic acid (**1**) and vanil-lic acid (**2**) may promote the movement of MEF cells.

In the present study, we aimed to evaluate the wound-healing properties of the isolated compounds from vinegar–egg. Our findings suggest that these compounds effectively promote the epithelialization stage of wound healing, the second stage in the four-phase wound-healing process, which includes inflammation, epithelialization, proliferation, and maturation [28]. The examined compounds facilitated wound healing by enhancing cell mobility and invasion without affecting cell proliferation, thereby playing a crucial role in barrier maintenance and re-epithelialization following injury.

To repair damaged skin, intercellular junctions must be loosened, and the activation of FAK and MMPs plays a crucial role in this process [54,55]. In this study, we observed that the tested compounds increased cell migration in a concentration-dependent manner by inducing FAK phosphorylation. The activation of FAK triggers downstream signaling pathways, such as MAPK. MAPKs are serine-threonine protein kinases that play a critical role in signal transduction from the cytoplasm to the nucleus and regulate various cellular functions, including differentiation, proliferation, development, inflammatory responses, apoptosis, and oxidative stress [18,19,28]. Similarly to EGF treatment, the tested compounds significantly enhanced p38 phosphorylation, leading to increased migration of MEF cell [56,57]. Furthermore, our results suggest that the tested compounds promote cell migration by modulating MMP2 expression. These findings imply that vinegar treatment, a simple and non-thermal process, can enhance the functional properties of egg-based products while preserving their nutritional composition. This strategy is consistent with current trends in the food industry that emphasize the development of minimally processed, health-promoting functional foods. Furthermore, the identification of safe, cost-effective, and biologically active compounds from widely consumed food materials opens new avenues for the formulation of value-added products, functional ingredients, and nutraceuticals. Leveraging readily available raw materials in such innovative ways holds significant potential to improve production efficiency and diversify product offerings within the functional food sector.

## 4. Conclusions

Natural products have long been used as relatively inexpensive and generally safe options for the prevention and treatment of diseases. In this context, this work set out to identify and characterize the bioactive phenolic compounds present in vinegar–egg and to evaluate their wound-healing potential. As a result, we investigated the effects of 4-hydroxy-benzoic acid (**1**) and vanillic acid (**2**) on wound healing in MEF cells. In our comparative in vitro assays, compounds **3** (methyl syringate) and **4** (leptosperin) were also tested at identical concentrations but demonstrated only limited wound-healing activity. This differential efficacy underscores the superior potency of 4-hydroxybenzoic acid and vanillic acid in promoting cell migration, and justifies our emphasis on these two phenolics for further evaluation. The active compounds, 4-hydroxy-benzoic acid (**1**) and vanillic acid (**2**), evaluated in this study were significantly more cost-effective than EGF while exhibiting similar effects. Cell viability assays demonstrated that concentrations of up to 100 μM of these compounds did not significantly affect cell viability or proliferation. However, scratch wound healing assays revealed that both compounds enhanced MEF cell wound closure. Live cell analysis using HoloMonitor further confirmed that individual cells treated with these compounds exhibited increased motility compared to the DMSO-treated control group. Transwell chamber invasion assays also demonstrated that 4-hydroxy-benzoic acid (**1**) and vanillic acid (**2**) enhanced the invasion ability of MEF cells, thereby facilitating wound closure. Additionally, we evaluated MMP2 expression and MAPK phosphorylation levels in the presence and absence of 4-hydroxy-benzoic acid (**1**) and vanillic acid (**2**). Our data indicate that treatment with these compounds increased the phosphorylation levels of FAK and p38. Collectively, these findings suggest that 4-hydroxy-benzoic acid (**1**) and vanillic acid (**2**) may serve as promising agents for accelerating wound healing by promoting cell migration and invasion through FAK-MAPK signaling and MMP2 modulation. To the best of our knowledge, this is the first report to isolate and characterize phenolic compounds from vinegar–egg and to demonstrate their wound-healing efficacy via modulation of focal adhesion and MAPK signaling pathways. This work not only provides a mechanistic explanation for the traditional use of vinegar–egg but also introduces a novel strategy for generating functional ingredients from safe and widely available food matrices.

## Data Availability

The original contributions presented in this study are included in the article/Supplementary Materials. Further inquiries can be directed to the corresponding authors.

## Supplementary Materials

The following are available online at https://www.mdpi.com/article/10.3390/nu17162584/s1; Figure S1: The ^1^H NMR spectrum of **1** (CD_3_OD, 850 MHz); Figure S2: The ESI-MS data of **1**; Figure S3: The ^1^H NMR spectrum of **2** (CD_3_OD, 850 MHz); Figure S4: The ESI-MS data of **2**; Figure S5: The ^1^H NMR spectrum of **3** (CD_3_OD, 850 MHz); Figure S6: The ESI-MS data of **3**; Figure S7: The ^1^H NMR spectrum of **4** (CD_3_OD, 850 MHz); Figure S8: The ESI-MS data of **4**; General Experimental Procedures; Cell motility videos for compounds **1**–**4**; Video File S1: Effects of DMSO on the motility of MEF cells in time-lapse cell tracking videos; Video File S2: Effects of **4**-Hydroxy-benzoic acid on the motility of MEF cells in time-lapse cell tracking videos; Video File S3: Effects of vanillic acid on the motility of MEF cells in time-lapse cell tracking videos; Video File S4: Effects of methyl syringate on the motility of MEF cells in time-lapse cell tracking videos; Video File S5: Effects of leptosperin on the motility of MEF cells in time-lapse cell tracking videos.
