# Supplementary material for: Bioactive Phenolics from Vinegar–Egg Accelerates Acute Wound Healing by Activation of Focal Adhesion and Mitogen-Activated Protein Kinase Signaling
Source: Nutrients. 2025 Aug 8;17(16):2584. doi: 10.3390/nu17162584 (PMC12389561; doi:10.3390/nu17162584)
Supplement: Supplementary file 1 [file nutrients-17-02584-s001.zip › nutrients-3766656-supplementary.pdf]

Supplementary Materials

Bioactive phenolics from vinegar-egg accelerates acute wound healing by a ctivation of focal adhesion and mitogen activated protein kinase signaling

Taehoon Oh [1,2,†], Chan Hee Cho [3,†], Su Cheol Baek [3], Mun Seok Jo [3], Woo Bong Kang [4], Yun Seok Kang [4], Sung-Kyun Ko [1,5,*] and Ki Hyun Kim [3,*]

[1] Chemical Biology Research Center, Korea Research Institute of Bioscience and Biotechnology (KRIBB), Cheongju 28116, Republic of Korea

[2] College of Pharmacy, Chungbuk National University, Cheongju, Chungbuk 28160, Republic of Korea

[3] School of Pharmacy, Sungkyunkwan University, Suwon 16419, Republic of Korea

[4] Crystal Insul Bio., Paju 10930, Republic of Korea

[5] KRIBB School of Bioscience, Korea University of Science and Technology (UST), Daejeon 34141, Republic of Korea

[†] These authors contributed equally

* Corresponding authors

* Sung-Kyun Ko Ph.D. - Chemical Biology Research Center, Korea Research Institute of Bioscience and Biotechnology (KRIBB), Cheongju 28116, Republic of Korea; Tel: +82-43-240-6146, Fax: +82-43-240-6169, Email address: ksk1230@kribb.re.kr

* Ki Hyun Kim Ph.D. - School of Pharmacy, Sungkyunkwan University, Suwon 16419, Republic of Korea; Tel: +82-31-290-7700; Fax: +82-31-290-7730; E-mail: khkim83@skku.edu

Supporting Information Contents

Figure S1. The ^{1}H NMR spectrum of **1** (CD_3OD, 850 MHz) S3

Figure S2. The ESI-MS data of **1** S4

Figure S3. The ^{1}H NMR spectrum of **2** (CD_3OD, 850 MHz) S5

Figure S4. The ESI-MS data of **2** S6

Figure S5. The ^{1}H NMR spectrum of **3** (CD_3OD, 850 MHz) S7

Figure S6. The ESI-MS data of **3** S8

Figure S7. The ^{1}H NMR spectrum of **4** (CD_3OD, 850 MHz) S9

Figure S8. The ESI-MS data of **4** S10

General Experimental Procedures S11

Video File S1. Effects of DMSO on the motility of MEF cells in time-lapse cell tracking videos

Video File S2. Effects of 4-Hydroxy-benzoic acid on the motility of MEF cells in time-lapse cell tracking videos

Video File S3. Effects of vanillic acid on the motility of MEF cells in time-lapse cell tracking videos

Video File S4. Effects of methyl syringate on the motility of MEF cells in time-lapse cell tracking videos

Video File S5. Effects of leptosperin on the motility of MEF cells in time-lapse cell tracking videos

Figure S1. The ^1H NMR spectrum of **1** (CD_3OD, 850 MHz)

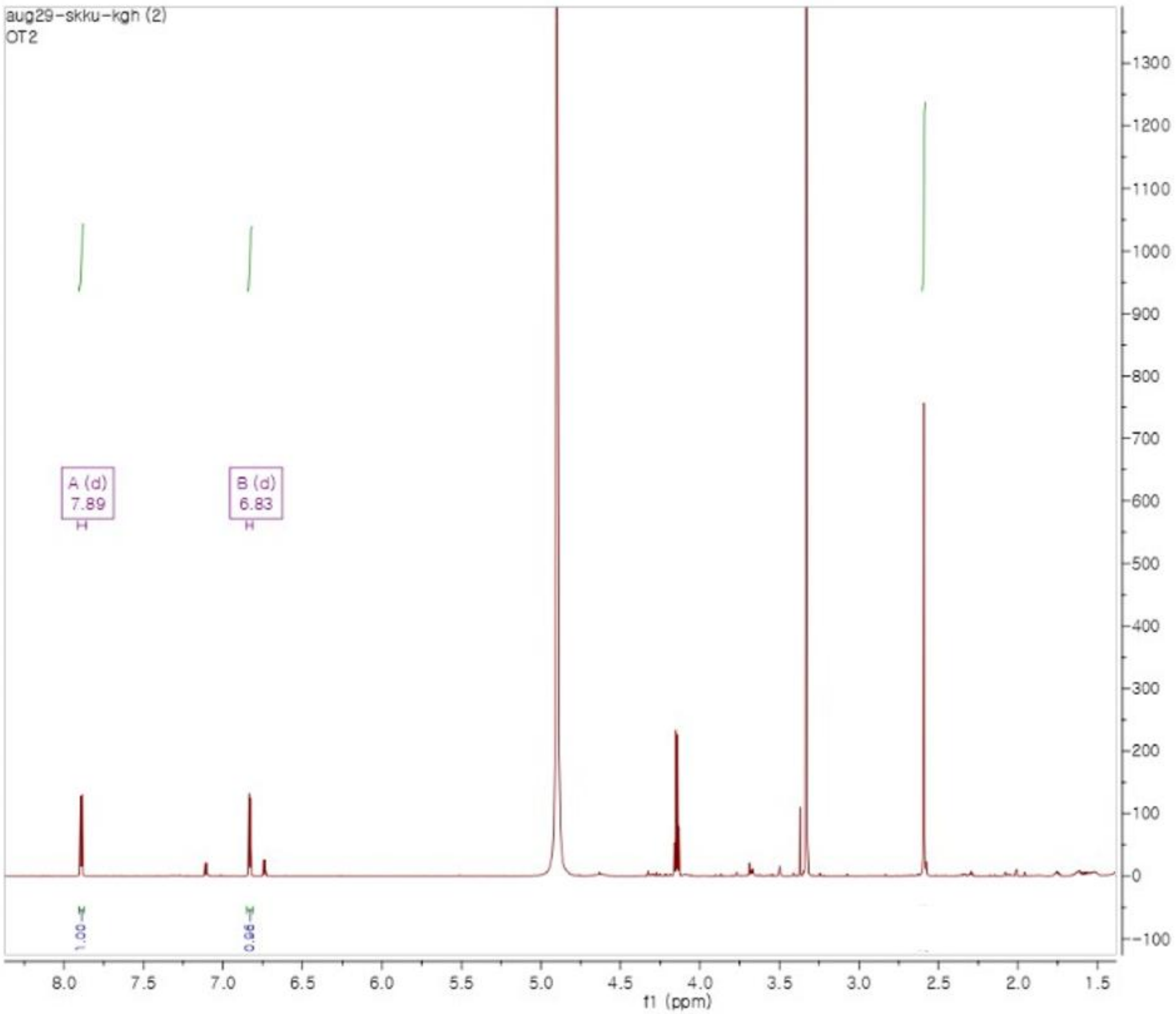

Figure S2. The ESI-MS data of **1**

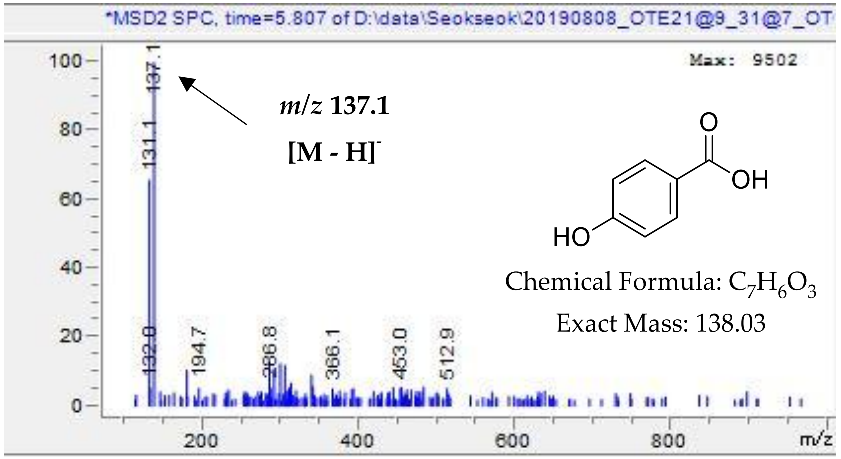

Figure S3. The ^{1}H NMR spectrum of **2** (CD_3OD, 850 MHz)

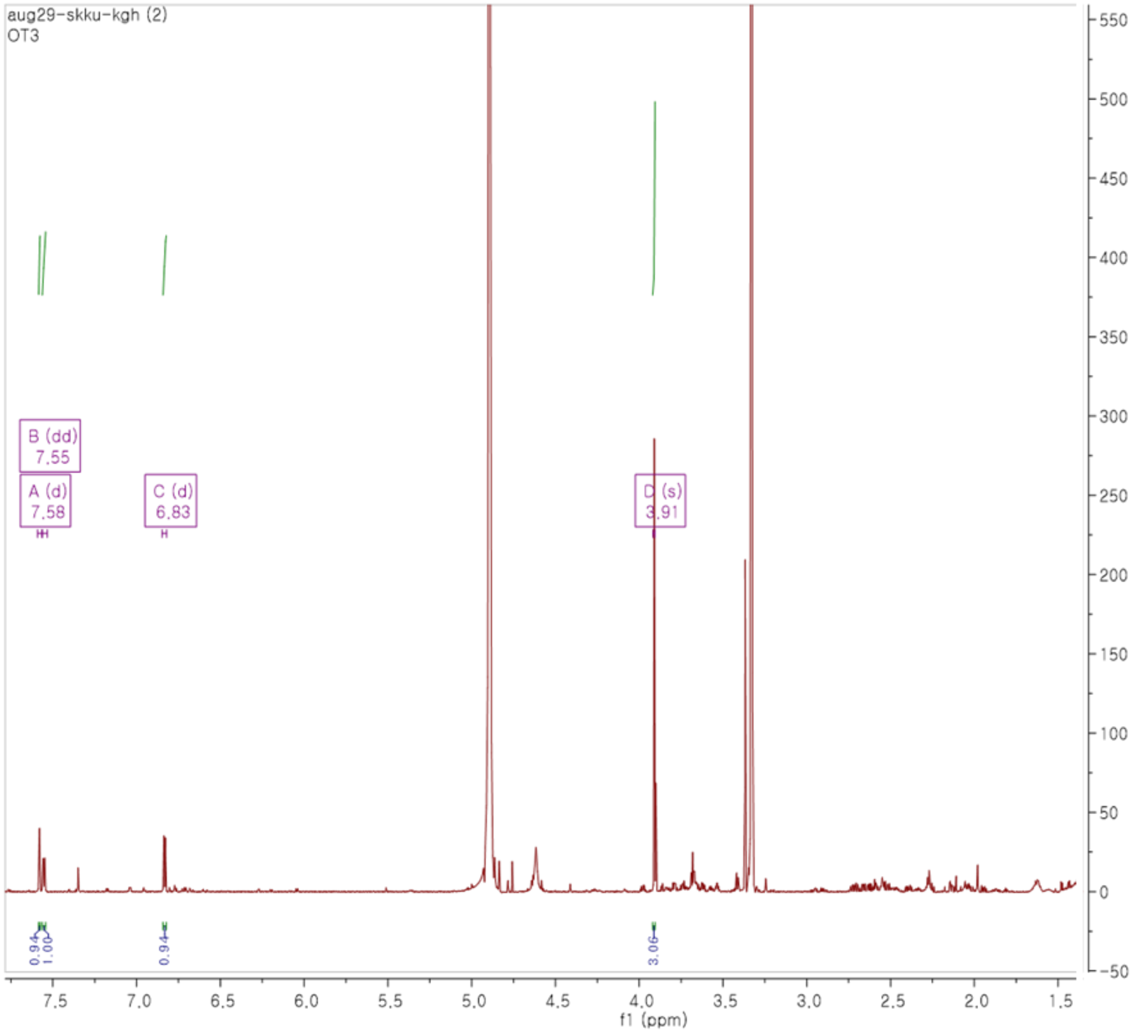

Figure S4. The ESI-MS data of **2**

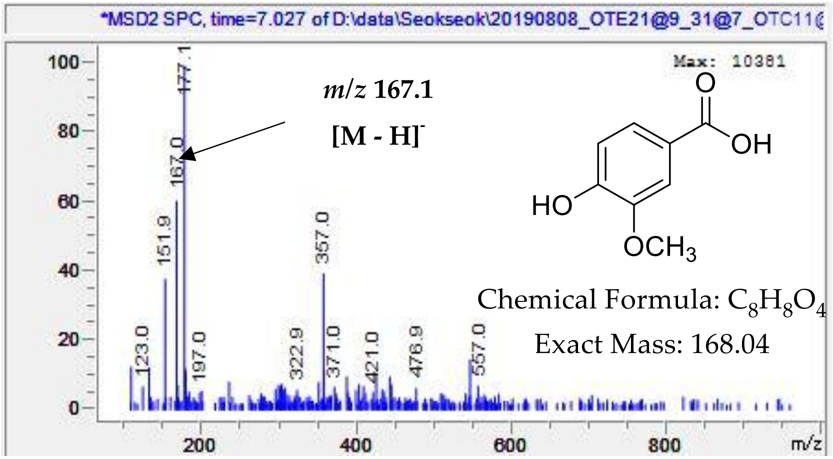

Figure S5. The ^{1}H NMR spectrum of **3** (CD_3OD, 850 MHz)

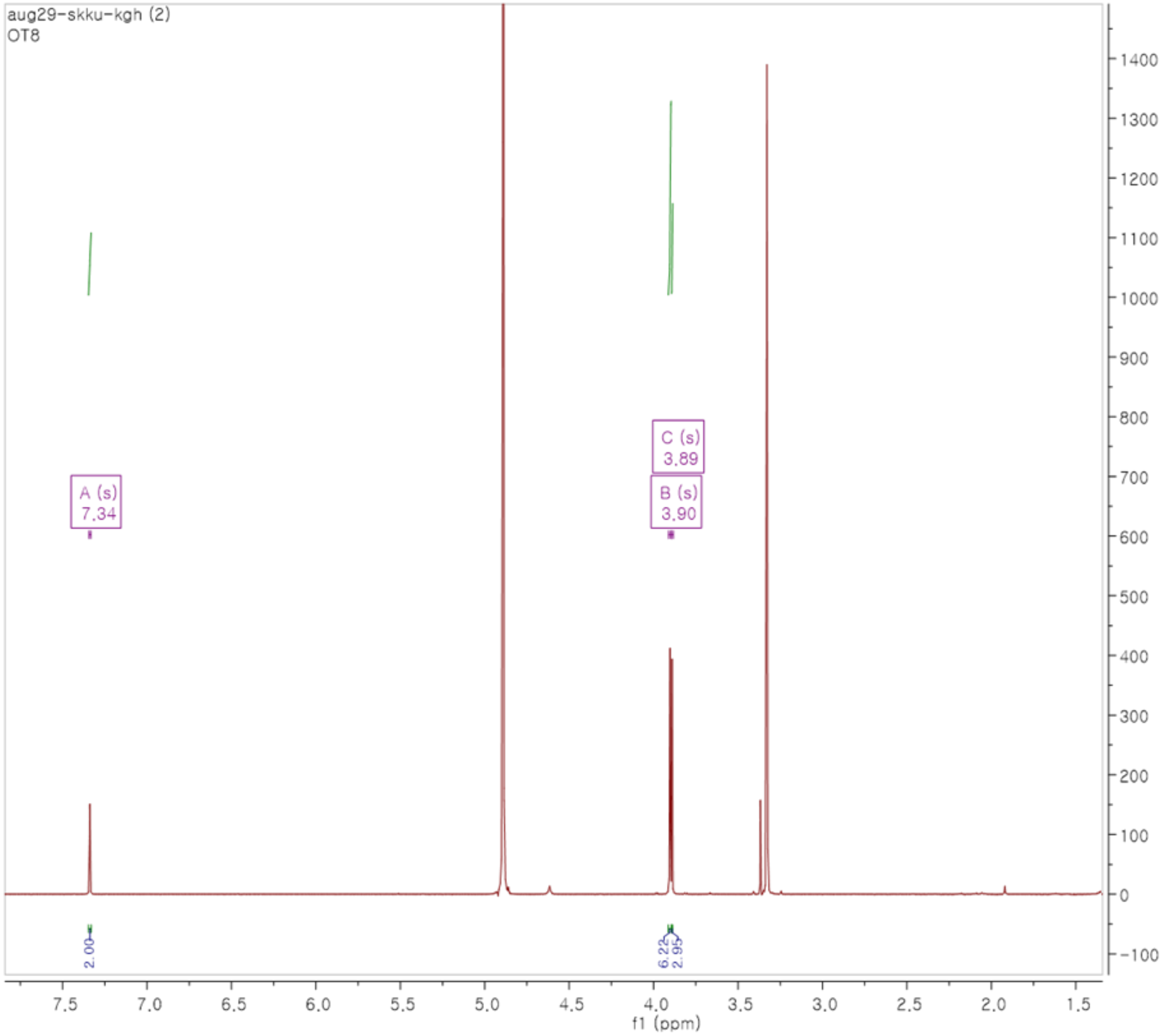

Figure S6. The ESI-MS data of **3**

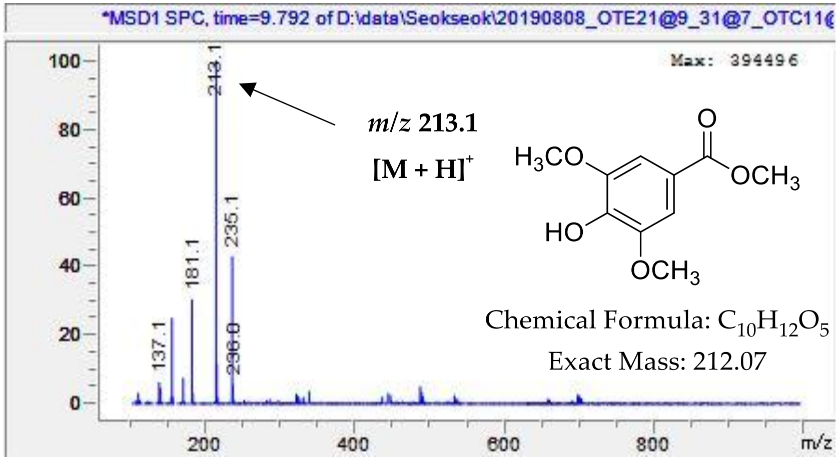

Figure S7. The ^{1}H NMR spectrum of **4** (CD_3OD, 850 MHz)

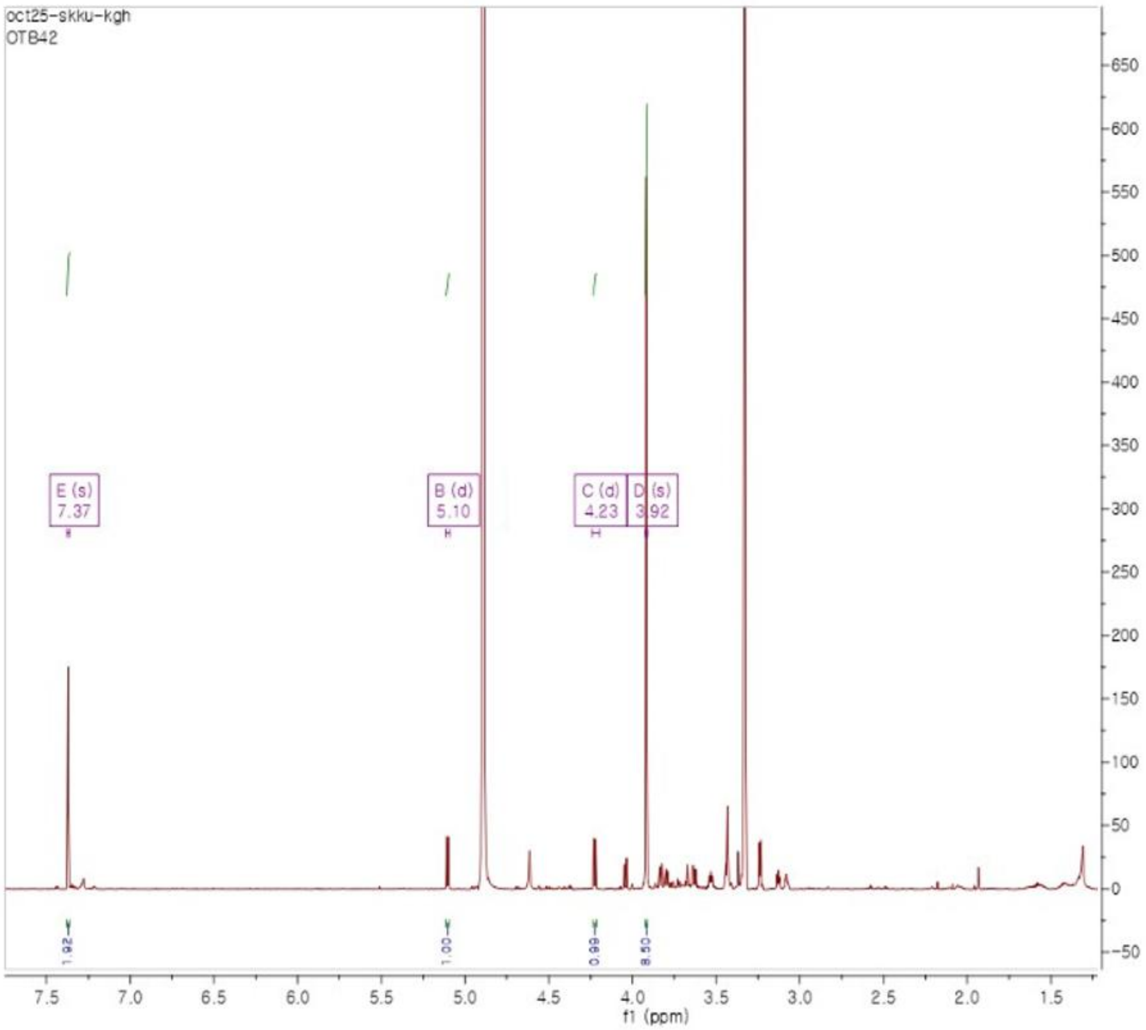

Figure S8. The ESI-MS data of **4**

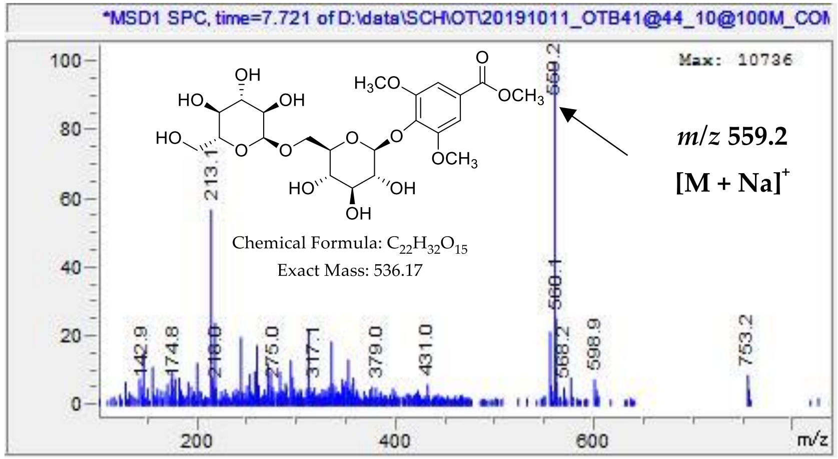

General Experimental Procedures

Optical rotation measurements were obtained using a Jasco P-1020 polarimeter (Jasco, Easton, MD, USA). Nuclear magnetic resonance (NMR) spectra were recorded on a Bruker AVANCE III HD 850 NMR spectrometer at 850 MHz for ^{1}H NMR and 212.5 MHz for ^{13}C NMR, with chemical shifts reported in ppm (δ). Semi-preparative HPLC was performed using a Waters HPLC system equipped with 996 photodiode array detectors and a Waters 1525 binary HPLC pump (Waters Corporation, Milford, CT, USA). LC/MS analysis was conducted on an Agilent 1200 Series HPLC system (Agilent Technologies, Santa Clara, CA, USA) equipped with a diode array detector and a 6130 Series ESI mass spectrometer, using an analytical Kinetex C18 100 Å column (100 mm × 2.1 mm i.d., 5 μm) (Phenomenex, Tor-rance, CA, USA). For thin-layer chromatography (TLC), Merck precoated silica gel F_{254} plates and RP-18 F_{254}s plates were used. Spots were detected under UV light or by heating after spraying with anisaldehyde-sulfuric acid.
